# Supplementary material for: Prevalence and determinants of anemia among HIV-positive children in central Uganda: A cross sectional study
Source: PLoS One. 2025 Nov 4;20(11):e0333825. doi: 10.1371/journal.pone.0333825 (PMC12585094; doi:10.1371/journal.pone.0333825)
Supplement: Attachment — Submitted filename: Response to reviewers.docx [file pone.0333825.s003.docx]

Response to Reviewers

| Comments given by | Area of comments | Comment given | Response given by Authors | Remark |
| --- | --- | --- | --- | --- |
| Reviewer  Reviewer  Reviewer | Title  Abstract  Abstract | The title in the manuscript draft ("Prevalence and Factors Associated With Anemia among HIV-Positive Children in Central Uganda: A Cross-sectional study") differs slightly from the title in the cover letter ("Prevalence and Factors Associated with Anemia among HIV-Positive Children at Kayunga Regional Referral Hospital, Uganda"). Ensure consistency throughout the document. | The title has been amended to reflect the scope of the study more clearly. The new title is: Prevalence and Factors Associated with Anemia among HIV-Positive Children in Central Uganda: A Cross-Sectional Study | Accepted and corrected |
|  |  | the sentence “Anemia was diagnosed if the hemoglobin level was below 11 g/dl…” should be rephrased for clarity | this has been rephrased and clearly stated (The diagnosis of anaemia was made if the haemoglobin level was less than 11g/dl) | Accepted and corrected |
|  |  | The phrase “lower education level of the caretaker” lacks specificity. Clarify whether this refers to primary education or no formal education. | this has been clarified and used primary level of education and non formal education were independently associated with anemia | Accepted and corrected **;** |
| Reviewer | Introduction | Global vs. Local Context: The introduction provides global and regional data well but could more explicitly state how this study fills a gap in the literature specific to Kayunga, beyond other Ugandan regions like Mbarara or Mulago. | This has been revised | Accepted and corrected |
| Reviewer | **Methodology**  Sampling Justification: | Please justify why a sample size of 384 was chosen (e.g., power calculation). Also clarify if this number was based on expected effect size or previous prevalence estimates. | sample size calculation was used KishLeslie formula to get sample size while using previous prevalence estimated and chosen power calculation | Accepted and corrected |
| Reviewer | **Methodology**  Ethical Statement  **Methodology**  Data Collection Tools:  **Methodology**  Data analysis: | It would enhance transparency to mention that written informed consent was obtained from caregivers and assent from older children in the Methods section, not only in the ethics statement.  Were the structured questionnaires pilot-tested before use? If so, include this information to strengthen methodological rigor.  Mentzer Index Calculation: Define the Mentzer index formula (MCV/RBC ratio) in the Methods section for readers unfamiliar with it.  Statistical Analysis Software Mention: While SPSS was used for logistic regression, STATA was mentioned later for data analysis. Clarify which software was primarily used and for what parts. | this has been mentioned in method section  yes, this has been stated  this has been defined in the method section  this has been corrected and SPSS was used for logistic regression | Accepted and corrected |
| Reviewer | **Results** | Severe Stunting/Wasting Definitions: Clarify how stunting and wasting were classified as "severe" were these based on WHO Z-scores (< -3 SD)? This should be clearly stated.  Binary Logistic Regression Threshold: The authors used a p-value ≤ 0.2 in bivariable analysis as a cutoff for inclusion in multivariable analysis. Is there a reference supporting this threshold?  Multivariable Model Fit: Was the model’s goodness-of-fit tested (e.g., Hosmer-Lemeshow test)? Include this to validate the logistic regression results. | this has been clearly stated and mention in the method section that has been used WHO growth chart (WHO Z-scores (<-3SD)  We did not utilize a p-value threshold of less than 0.25 for making statistical assertions. Instead, we employed this threshold as a screening criterion for selecting variables in multivariable logistic regression, aiming to streamline the candidate variable pool. For determining statistical significance, we adhered to the conventional threshold of less than 0.05. | Accepted and corrected  Accepted and corrected |
| Reviewer | **Discussion/**  **Conclusion** | Comparison with Previous Studies: The discussion compares findings with studies in Ethiopia, Nigeria, and India, but less attention is given to local Ugandan contexts. Expand on how findings differ or align with national-level policies or programs. | This has been revised | Accepted and corrected |
