# Supplementary material for: Prevalence and determinants of anemia among HIV-positive children in central Uganda: A cross sectional study
Source: PLoS One. 2025 Nov 4;20(11):e0333825. doi: 10.1371/journal.pone.0333825 (PMC12585094; doi:10.1371/journal.pone.0333825)
Supplement: Attachment — Submitted filename: Reviewer comments Anemia.docx [file pone.0333825.s001.docx]

Thank you for providing me with the opportunity to review the manuscript titled "Prevalence and Factors Associated with Anemia among HIV-Positive Children in Central Uganda: A Cross-sectional Study." While the study is important for researchers and stakeholders, I have a concern that could help improve the manuscript:

**General Comments and Suggestions**

1. The title is informative but can be slightly shortened without losing meaning. Consider:

“Prevalence and Determinants of Anemia among HIV-Positive Children in Central Uganda: A Cross-Sectional Study.”

**Abstract**

2. In the abstract, the sentence “Anemia was diagnosed if the hemoglobin level was below 11 g/dl…” should be rephrased for clarity.

3. The phrase “lower education level of the caretaker” lacks specificity. Clarify whether this refers to primary education or no formal education.

4. Keywords: Consider adding “pediatrics” , “nutritional deficiency” , and “cross-sectional study” to improve discoverability.

**Introduction**

5. Global vs. Local Context: The introduction provides global and regional data well but could more explicitly state how this study fills a gap in the literature specific to Kayunga, beyond other Ugandan regions like Mbarara or Mulago.

**Methodology**

6. Sampling Justification: Please justify why a sample size of 384 was chosen (e.g., power calculation). Also clarify if this number was based on expected effect size or previous prevalence estimates.

7. Ethical Statement: It would enhance transparency to mention that written informed consent was obtained from caregivers and assent from older children in the Methods section, not only in the ethics statement.

8. Data Collection Tools: Were the structured questionnaires pilot-tested before use? If so, include this information to strengthen methodological rigor.

9. Mentzer Index Calculation: Define the Mentzer index formula (MCV/RBC ratio) in the Methods section for readers unfamiliar with it.

10. Statistical Analysis Software Mention: While SPSS was used for logistic regression, STATA was mentioned later for data analysis. Clarify which software was primarily used and for what parts.

**Results Section Feedback**

11. Table Formatting: Tables are informative but need clearer formatting (e.g., consistent decimal places, alignment, bold headers). Consider using table footnotes where necessary.

12. Iron Deficiency Anemia Interpretation: The finding that 54.1% of anemic children had iron deficiency may be overestimated if relying solely on Mentzer index. Discuss limitations of using this index instead of serum ferritin or other lab markers.

13. Severe Stunting/Wasting Definitions: Clarify how stunting and wasting were classified as "severe" were these based on WHO Z-scores (< -3 SD)? This should be clearly stated.

14. Binary Logistic Regression Threshold: The authors used a p-value ≤ 0.2 in bivariable analysis as a cutoff for inclusion in multivariable analysis. Is there a reference supporting this threshold?

15. Multivariable Model Fit: Was the model’s goodness-of-fit tested (e.g., Hosmer-Lemeshow test)? Include this to validate the logistic regression results.

**Discussion and Conclusion**

16. Comparison with Previous Studies: The discussion compares findings with studies in Ethiopia, Nigeria, and India, but less attention is given to local Ugandan contexts. Expand on how findings differ or align with national-level policies or programs.

17. Limitations Generalizability: The limitation regarding generalizability due to facility-based sampling is noted. Consider discussing potential selection bias or implications for broader Ugandan populations.

18. Confounding Variables: Did the authors consider adjusting for variables such as dietary intake, micronutrient supplementation, or malaria status in the final model? These could confound the observed associations.

19. Public Health Implications:The conclusion mentions routine screening and caregiver education. Expand on feasible interventions tailored to resource-limited settings like Kayunga (e.g., community health worker involvement).

20. Future Research Directions: Recommend future longitudinal studies to explore causal relationships between HIV progression and anemia. Also, suggest investigating the impact of ART regimens on hematologic parameters.

**Minor Editorial Suggestions**

Ensure consistency in terminology (e.g., “HIV-positive children” vs. “children living with HIV”).

Check all acronyms for first-time definitions (e.g., MI = Mentzer Index).

Avoid repetitive phrases like “in this study” throughout the Discussion.

Use active voice where possible for better readability.

Review figures for resolution and clarity (especially Figure 1 in PDF format)
